# Ectoparasites of European hedgehogs (*Erinaceus europaeus*) in Germany and their health impact

**DOI:** 10.1186/s13071-023-06081-9

**Published:** 2024-01-02

**Authors:** Karolin Schütte, Andrea Springer, Florian Brandes, Maximilian Reuschel, Michael Fehr, Christina Strube

**Affiliations:** 1 Institute for Parasitology, Centre for Infection Medicine, University of Veterinary Medicine Hannover, Hanover, Germany; 2 Wildlife Rescue and Conservation Center Sachsenhagen, Sachsenhagen, Germany; 3 Department of Small Mammal, Reptile and Avian Diseases, University of Veterinary Medicine Hannover, Hanover, Germany

**Keywords:** Ticks, Fleas, Mites, *Ixodes ricinus*, *Ixodes hexagonus*, *Archaeopsylla erinacei*, *Caparinia tripilis*, Infestation intensity

## Abstract

**Background:**

The European hedgehog (*Erinaceus europaeus*) is known for high levels of ectoparasitism that not only represents a health risk for the animals themselves, but also for pet animals and humans as hedgehogs are frequently taken into human care. In the present study, patterns of ectoparasite infestation were assessed in hedgehogs taken into care at northern German animal rehabilitation centres.

**Methods:**

Ectoparasites (ticks, fleas and mites) of 498 hedgehogs were collected over a period of 3 years from July 2018 to May 2021. Species were identified based on morphological characteristics and also via amplification and sequencing of the partial cytochrome c oxidase subunit 2 (COX-2) gene for fleas of the family Ceratophyllidae. Seasonal changes in infestation patterns as well as correlations with animal age, body weight and health status were assessed using generalised linear models.

**Results:**

Infestation with ticks, fleas and mites occurred throughout the year. Overall, 86.5% (431/498) of the examined hedgehogs were infested with ticks, 91.4% (455/498) with fleas and 17.7% (88/498) with mites*.* *Ixodes ricinus* and *Ixodes hexagonus*/*Ixodes canisuga* were the most common tick species detected, with the additional occurrence of one* Ixodes frontalis.* Significant seasonal changes were observed for *I. ricinus*, but not for *I. hexagonus*/*I. canisuga*. Additionally, *I. ricinus* nymph prevalence declined significantly as of 2020, probably as a consequence of the climate change-related drought as of 2018. In hedgehogs with flea infestations, *Archaeopsylla erinacei*, *Ceratophyllus sciurorum*, *Nosopsyllus fasciatus* and *Ctenocephalides felis* were identified. In all cases of mite infestation, *Caparinia tripilis* was detected, in addition to specimens of the family Macronyssidae and free-living mites of the family Acaridae. Statistical analyses showed correlations regarding the factors month, year, body weight and age, but no correlation was evident regarding the health status of the animals.

**Conclusions:**

With a detected infestation rate of 98.6%, almost all of the examined hedgehogs were infested with at least one ectoparasite species. The seasonal activity patterns of the different ectoparasite species together with the complex annual cycle of hedgehogs lead to different seasonal patterns in ectoparasite prevalence and infestation intensities. Due to the risk of transmission of zoonotic pathogens as well as the possible negative impact on the host itself, hedgehogs should be treated against ectoparasites when taken into care facilities.

**Graphical Abstract:**

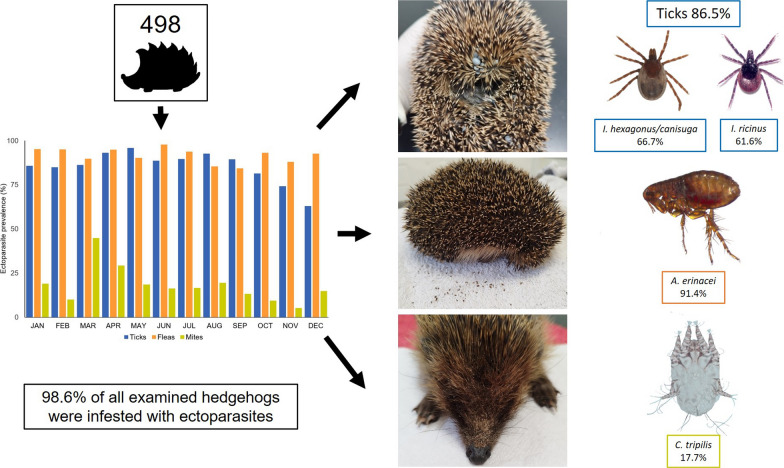

## Background

The European hedgehog (*Erinaceus europaeus*) is a synanthropic species frequently taken into human care, either in animal facilities or private homes, and presented to veterinary practices in central Europe. Hedgehogs often carry high ectoparasite loads [1, 2], which can negatively impact their own health due to blood loss, skin irritation, pruritus and inflammation [3]. However, hedgehogs also represent a public health risk due to the possible transmission of zoonotic vector-borne pathogens, such *Borrelia* spp., *Anaplasma phagocytophilum* and tick-borne encephalitis virus in the case of ticks, and *Bartonella* spp. in the case of fleas, among others [4, 5]. The epidemiological importance of hedgehogs as reservoirs of these pathogens is currently unknown, but *Borrelia* spp. and *A. phagocytophilum* have been detected in blood and/or tissue samples and ticks collected from European hedgehogs [6–8]. Furthermore, Hornok et al. [9] detected *Rickettsia* spp., *Bartonella henselae* and hemotropic *Mycoplasma* spp. in the hedgehog flea *Archeopsylla erinacei* collected from Northern white-breasted hedgehogs (*Erinaceus roumanicus*). In addition, *Bartonella* spp. have also been found in tissue samples of different hedgehog species [10].

The most frequent tick species infesting hedgehogs in central Europe are *Ixodes ricinus* and *Ixodes hexagonus* [11, 12], although *Haemaphysalis erinacei*, *Rhipicephalus turanicus* and *Rhipicephalus sanguineus* sensu lato have also been found on European hedgehogs in the southern part of their range [13, 14]. While *I. ricinus* is a generalist tick, which infests a large variety of hosts, including humans, *I. hexagonus* is a specialist tick with a nest-adapted lifestyle [15]. Occasionally, *I. hexagonus* can also be found on different hosts, especially mustelids [16], and also on dogs and cats [17]. Recently, Probst et al. [18] showed that *I. hexagonus* is the second most common tick species infesting cats and the third most common tick species infesting dogs in Germany.

Flea infestation of hedgehogs is most often due to the hedgehog-specific species *A. erinacei*, whereas *Nosopsyllus fasciatus, Ctenocephalides felis, Ceratophyllus gallinae* and *Ctenophthalmus* spp. are found more rarely [1, 14, 19–21]. Despite its rather high degree of host-specificity, *A. erinacei* can infest dogs and cats as well as humans upon contact with hedgehogs or their nests [20, 22–24].

In addition to tick and flea infestation, hedgehogs regularly suffer from dermatologic problems caused by mites. The most common mite species parasitising European hedgehogs is *Caparinia tripilis*, in addition to *Acarus nidicolus*, *Ornithonyssus* spp., *Demodex erinacei*, *Notoedres cati* and *Sarcoptes scabiei* [3, 13, 21, 25–27]. Transmission of mites may be facilitated by phoretic behaviour, which has been observed between *C. tripilis* and *A. erinacei* [13] as well as between nymphs of *A. nidicolus* and several flea species, including *A. erinacei* [28]. Unlike other mite species, which penetrate deeper into the skin, *C. tripilis* can be seen by the naked eye. It preferably infests the nasal bridge as well as the ears, leading to pruritus, secondary bacterial infections and otitis [3]. In severe cases, mites may cover the whole body. Because *C. tripilis* is considered to be a vector for *Trichophyton* spp., a secondary dermatophyte infection occurs frequently [29–31]. Due to the zoonotic potential of *Trichophyton* spp., *C. tripilis* infestation also poses a risk of dermatophyte transmission to humans when handling infested animals [32, 33]. Both mite infestation and dermatophyte infection often result in the loss of prickles and hair [3], and long recovery times as hedgehogs need their spines for defence.

Due to their synanthropic lifestyle, hedgehogs are often found and taken care of by humans, resulting in close contact and the possibility of zoonotic disease transmission. In urbanised areas, hedgehogs carry even higher ectoparasite burdens than in rural areas [11], possibly due to a higher population density, which facilitates transmission between individuals, especially if hedgehogs gather at anthropogenic food sources. Therefore, ectoparasites of hedgehogs not only represent a health risk for the animals themselves, but also for pet animals and humans. The aim of this study was to evaluate the infestation of European hedgehogs with ectoparasites (ticks, fleas and mites) and to assess seasonal infestation changes and possible correlations with different factors, including the health status of the animals.

## Methods

### Study animals

**Fig. 1:**
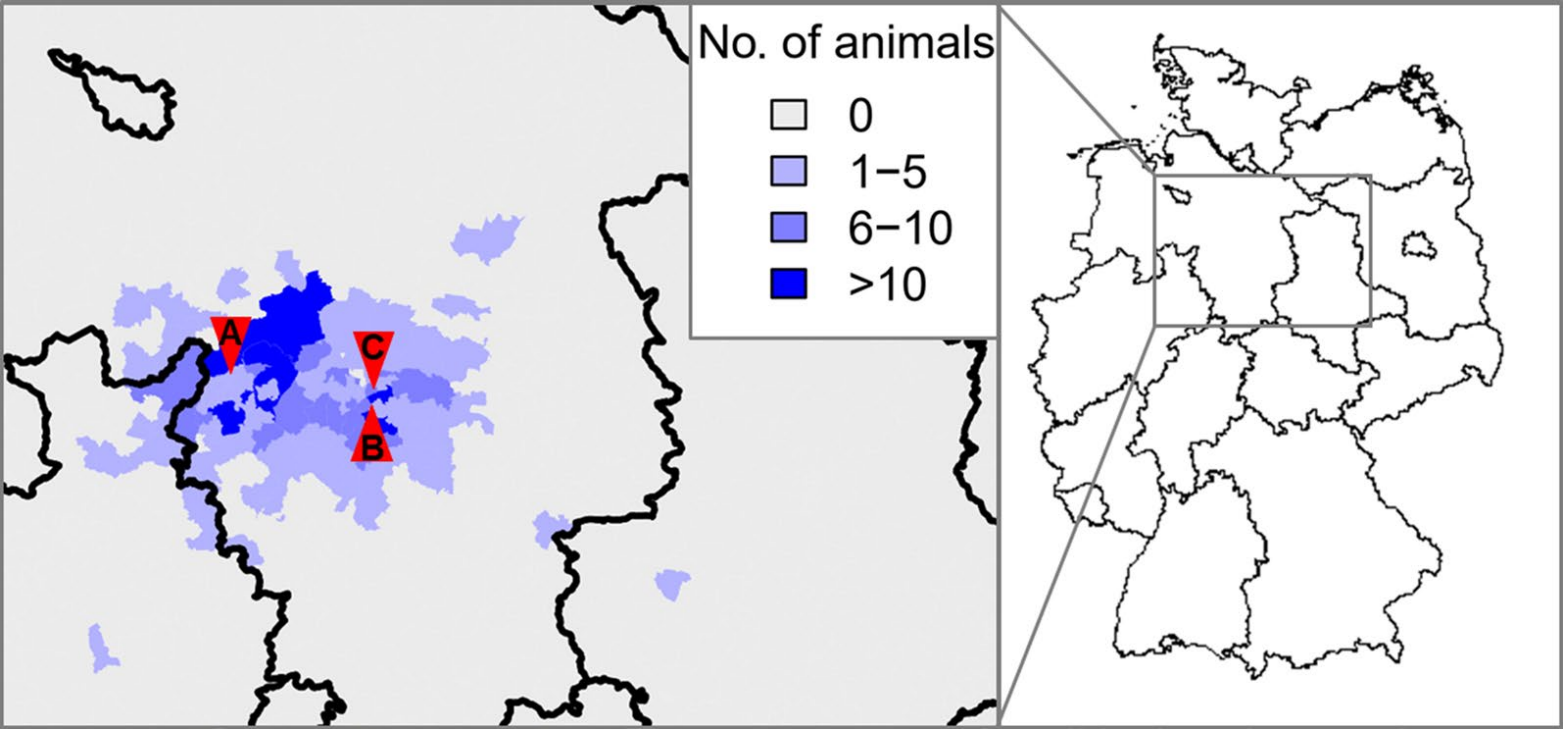
Origin of studied European hedgehogs (*Erinaceus europaeus*) (blue shading) and location of rehabilitation centres (red arrows) in the federal state of Lower Saxony, Germany. Uppercase A indicates the location of the Wildlife Rescue and Conservation Center (Sachsenhagen); uppercase B, *Aktiontier* Hedgehog Center (Laatzen); uppercase C, the Department of Small Mammal, Reptile and Avian Diseases of the University of Veterinary Medicine Hannover

In total, 498 European hedgehogs (*Erinaceus europaeus*) were examined over a period of 3 years from July 2018 to May 2021, with a break in December 2020 and January 2021. Sampling took place at three rehabilitation centres in the federal state of Lower Saxony, northern Germany: the Wildlife Rescue and Conservation Center (Sachsenhagen), the *Aktiontier* Hedgehog Center (Laatzen) and the Department of Small Mammal, Reptile and Avian Diseases of the University of Veterinary Medicine Hannover (Fig. 1). Hedgehogs were brought into the rehabilitation centres due to weakness, illness or injury. To record the origin of hedgehogs, the postal code of the finding site was noted. Hedgehog age was estimated by taking the body weight, body condition, teeth condition and season into account, and was classified as: (i) juvenile (suckling hedgehogs, still dependent on their mother); (ii) subadult (hedgehogs with an approximate age < 1 year); and (iii) adults (animals with an approximate age > 1 year). The health status was categorised on a scale of 0–2, with 0 indicating a normal health status (most of these animals were brought to the stations because of being supposedly too small); 1 indicating moderately impaired health (e.g. weakness, diarrhoea, hypothermia, dehydration, cough, minor injuries or conjunctivitis; with a maximum of 3 of these clinical signs); and 2 indicating severely impaired health (≥ 3 of the above-mentioned signs and/or serious injuries, abscesses, pneumonia, dyspnoea, myiasis). Dependent on the health status, the animals were later reintroduced into the wild, euthanised or they died during rehabilitation.

### Sample collection and morphological determination

Hedgehogs were examined for ectoparasites upon arrival at the respective centre as part of the routine medical procedure for rescued animals. Ectoparasite specimens were collected from the skin of the animals using a forceps, flea comb, adhesive tape or other tools. In the case of a high tick load, it was not possible to collect all ticks (especially larvae), as the lengthy sampling procedure would have been too stressful for the animal. Fleas were collected either during a short period of isoflurane anaesthesia, which is frequently necessary to fully examine a hedgehog, or after treatment with antiparasitics. Mites were collected using adhesive tape or forceps. All ectoparasites were stored at − 80 °C until identification by morphological characteristics using a (stereo-)microscope (models Zeiss Stemi SV11, Zeiss SteREO Discovery.V8 or Zeiss Primo Star; Carl Zeiss AG, Jena, Germany) and published keys [25, 34–38]. Nymphs and larvae of *I. hexagonus* and *I. canisuga* could not be differentiated by morphology with certainty and were therefore summarised as *I. hexagonus*/*I. canisuga*. With regard to fleas, up to 100 specimens per animal were morphologically identified and the remaining fleas were counted only. In two cases, the infestation intensity was so high that it was extrapolated by determining the average weight of 5 × 100 fleas and weighing the remaining fleas. Mites were embedded into Berlese solution (30 g Gummi arabicum, 50 ml Aqua Dest, 20 ml glycerin and 200 g crystalline chloral hydrate) and incubated for 4 h at 70 °C before morphological identification of male specimens.

### Molecular determination of Ceratophyllidae

As it was not possible to morphologically identify fleas of the family Ceratophyllidae to species level, they were subjected to molecular identification by amplification and sequencing of the partial cytochrome c oxidase subunit 2 (COX-2) gene. DNA was isolated from fleas by first grinding the entire or half of a flea in 90 or 45 µl DirectPCR Lysis Reagent (DirectPCR^®^ Lysis Reagent Cell; Peqlab, Erlangen, Germany) with a pestle, followed by the addition of 10 or 5 µl proteinase K, respectively (20 mg/ml; Macherey–Nagel GmbH & Co. KG, Dueren, Germany). Incubation at 55 °C for 14–16 h and subsequent inactivation for 45 min at 85 °C followed. A 780-bp fragment of the COX-2 gene was amplified using primers F-Leu and R-Lys [39]. The reaction was carried out in a final reaction volume of 50 μl containing 1.25 U (0.25 μl) DreamTaq polymerase, 5 μl 10× DreamTaq buffer (including 20 mM MgCl2) (Thermo Fisher Scientific, Waltham, MA, USA), 1 μl PCR nucleotide mix (0.2 mM each; Roti®^-^Mix PCR 3; Carl Roth GmbH + Co. KG, Karlsruhe, Germany), 5 μl of each primer (1 μM final concentration) and 2–5 μl template DNA. For amplification, an initial denaturation step at 95 °C for 3 min was followed by 40 cycles of denaturation at 95 °C for 30 s, annealing at 53 °C for 1 min and extension at 72 °C for 1 min; a final extension was performed at 72 °C for 7 min.

The amplicons were visualised on a GelRed®-supplemented (1:10,000; Biotium, Inc., Fremont, CA, USA) 1.5% agarose gel. Sanger sequencing was carried out at the Microsynth Seqlab Laboratories (Göttingen, Germany), and a BLAST (Basic local alignment search tool) search of obtained sequences was run against the NCBI (National Centre for Biotechnology Information) database.

### Statistical analysis

Statistical analyses were conducted in R v. 4.1.0 [40]. The prevalence of *I. ricinus* and *I. hexagonus/I. canisuga* was compared using the Chi-square test (*χ*^2^), while infestation intensity of these tick species was compared using the Wilcoxon signed rank test for paired data in cases of co-infested animals. Presence/absence data of *I. ricinus*, *I. hexagonus* and mites in relation to sampling month, sampling year, sex, age, body weight and health status were investigated using binomial generalised linear models (GLMs). Due to the high prevalence of flea infestation, no binomial GLMs for flea presence/absence were calculated due to the low informative value. For ticks, separate binomial GLMs were constructed for nymphs and adult ticks, in addition to total tick infestation. Further, total tick and flea infestation intensity was investigated via negative-binomial GLMs including the same predictor variables. Due to missing data, models were calculated for a subset of 388 animals. The models were compared to null models containing only the intercept in a likelihood ratio test (R function ANOVA, test = ´chisq´).

## Results

### Sampled animals

**Fig. 2:**
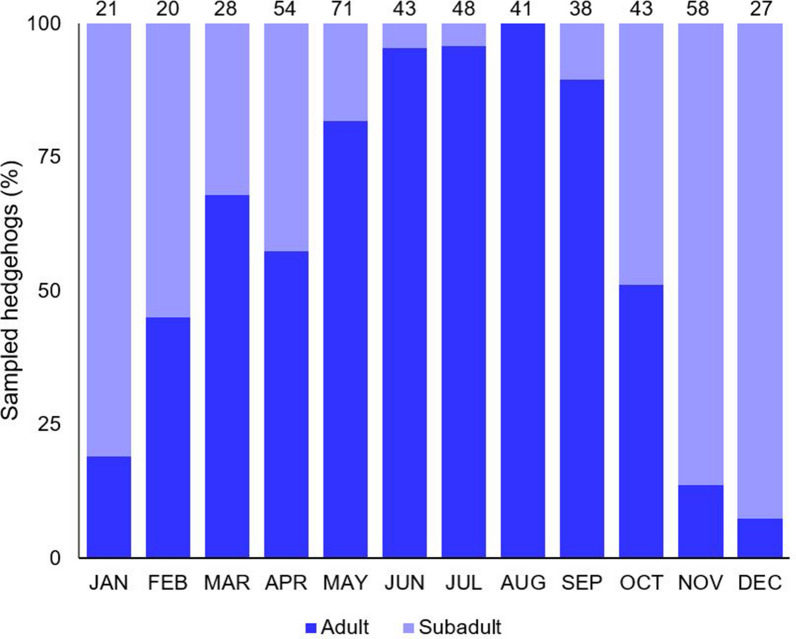
Monthly age distribution of sampled European hedgehogs. Total numbers of examined animals each month are shown above the bars. The graph includes the age of 492 hedgehogs as the age of 6 animals was not determined

In total, 498 hedgehogs were available for assessment in this study. The animals originated primarily from the federal state of Lower Saxony, with the addition of a few hedgehogs found in the federal states of North Rhine-Westphalia and Saxony-Anhalt (Fig. 1). Sex, age and weight distribution of the animals are shown in Table 1. Nearly equal numbers of male and female hedgehogs were sampled. The age of 492 hedgehogs could be determined, of which 64.0% (315/492) were adult and 36.0% (177/492) were subadult. The monthly age distribution is shown in Fig. 2. No juvenile hedgehogs were examined. Most of the adult animals came to the care stations with an impaired health status, predominantly due to a traumatic injury (33.7% [106/315]), which was less often observed in subadult hedgehogs (12.4% [22/177]). Adult hedgehogs also had to be euthanised more often (32.7% [103/315]) than subadults (7.3% [13/177]). Most hedgehogs were sampled during spring (31.7% [158/498]), summer (26.7% [133/498]) and autumn (27.9% [139/498]), while only few specimens were examined during winter (13.7% [68/498]).

**Table 1:** Key data on the European hedgehog (*Erinaceus europaeus*) specimens examined for ectoparasites

Data	Total hedgehogs,* n* (%)	Subadult hedgehogs,* n* (%)	Adult hedgehogs,* n* (%)	Undetermined age,* n* (%)
*Number*	498 (100)	177 (35.5)	315 (63.3)	6 (1.2)
*Sex*				
Male	237 (47.6)	80 (45.2)	155 (49.2)	2 (33.3)
Female	231 (46.4)	84 (47.5)	143 (45.4)	4 (66.7)
Not determined	30 (6.0)	13 (7.3)	17 (5.4)	0 (0.0)
*Weight*				
0–499 g	187 (37.6)	165 (93.2)	18 (5.7)	4 (66.7)
500–1000 g	251 (43.2)	12 (6.8)	237 (75.2)	2 (33.3)
> 1000 g	40 (8.0)	0 (0.0)	40 (12.7)	0 (0.0)
Not determined	20 (4.0)	0 (0.0)	20 (6.3)	0 (0.0)
*Sampling year (month)*				
2018 (July–Dec)	67 (13.5)	39 (22.0)	28 (8.9)	0 (0.0)
2019 (Jan–Dec)	215 (43.2)	91 (51.4)	121 (38.4)	3 (50.0)
2020 (Jan–Nov)	178 (35.7)	36 (20.3)	142 (45.1)	0 (0.0)
2021 (Feb–May)	38 (7.6)	11 (6.2)	24 (7.6)	3 (50.0)
*Health status*				
Not impaired	16 (3.2)	14 (7.9)	2 (0.6)	0 (0.0)
Moderately impaired	179 (35.9)	81 (45.8)	94 (29.8)	4 (66.7)
Severely impaired	249 (50.0)	40 (22.6)	207 (65.7)	2 (33.3)
Not determined	54 (10.8)	42 (23.7)	12 (3.8)	0 (0.0)
*Trauma*				
No	370 (74.3)	155 (87.6)	209 (66.3)	6 (100)
Yes	128 (25.7)	22 (12.4)	106 (33.7)	0 (0.0)
*Fate*				
Released	302 (60.6)	141 (79.7)	157 (49.8)	4 (66.7)
Euthanised	118 (23.7)	13 (7.3)	103 (32.7)	2 (33.3)
Died	77 (15.5)	23 (13.0)	54 (17.1)	0 (0.0)
Unknown	1 (0.2)	0 (0.0)	1 (0.3)	0 (0.0)

### Ectoparasite infestation

**Fig. 3:**
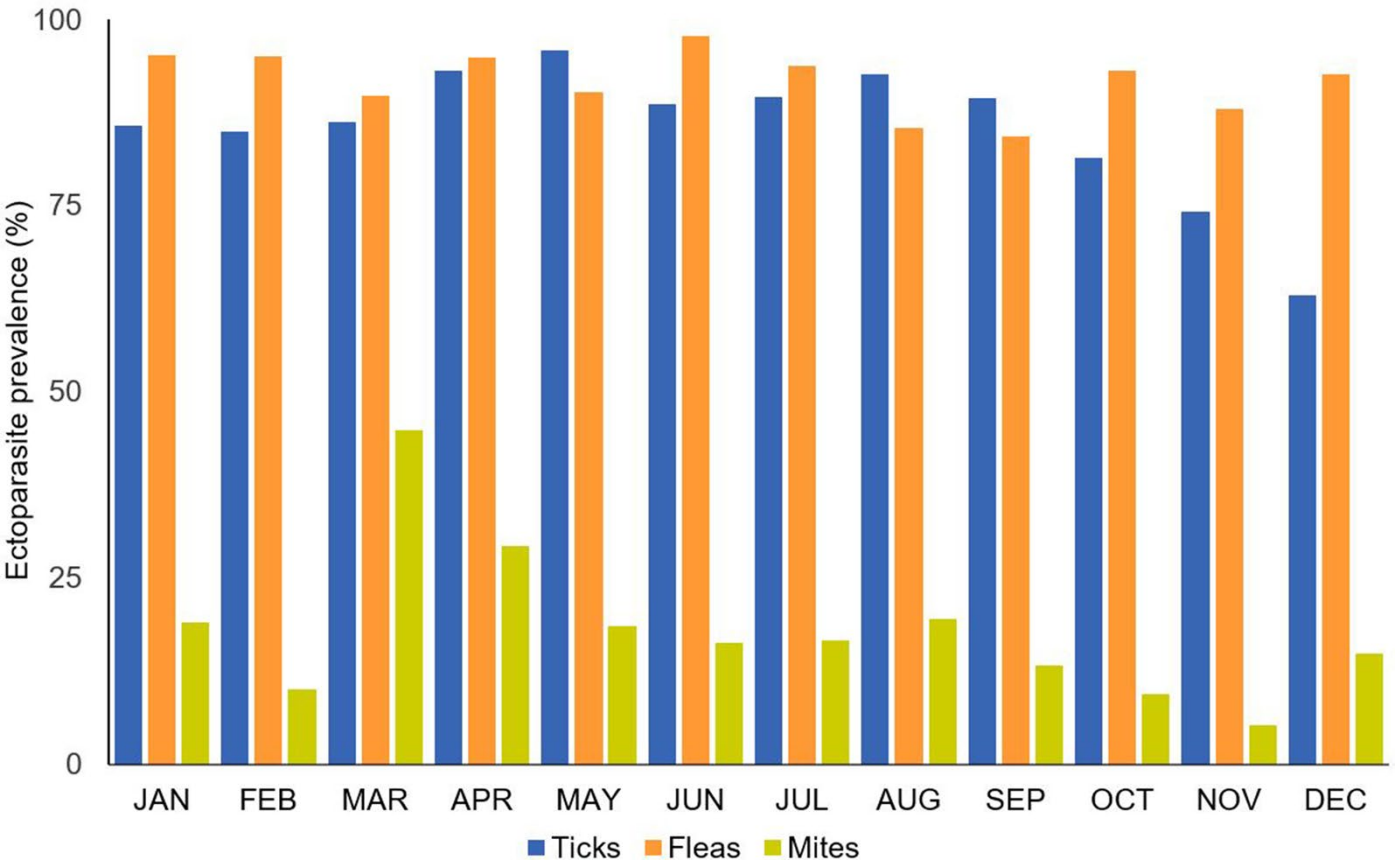
Seasonal distribution of tick, flea and mite prevalence among 498 European hedgehogs in northern Germany during the study period 2018–2021

Infestation with ticks, fleas and mites occurred throughout the year (Fig. 3). Overall, 86.5% (431/498) of the examined hedgehogs were infested with ticks, with 81.9% (145/177) of subadults infested, 89.2% (281/315) of adults infested and five of the six animals of undetermined age infested. Flea infestation occurred in 91.4% (455/498) of the hedgehogs, with 89.8% (159/177) of subadults infested, 92.1% (290/315) of adults infested and all six of the animals of undetermined age infested. Mite infestation was found in 17.7% (88/498) of the examined hedgehogs, with 5.6% (10/177) of subadults infested and 24.8% (78/315) of adults infested. Co-infestation was determined in 81.9% (408/498) of animals, with 64.7% (322/498) of hedgehogs harbouring ticks and fleas, 15.1% (75/498) harbouring ticks, fleas and mites, 2.0% (10/498) harboring fleas and mites and 0.2% (1/498) harbouring ticks and mites.

### Ticks

The examined hedgehogs harboured mainly ticks of the species *I. ricinus* and *I. hexagonus*/*I. canisuga*, with the additional occurrence of one *I. frontalis* female (Table 2). The prevalence of *I. hexagonus*/*I. canisuga* was slightly, but not significantly, higher than that of *I. ricinus* (66.7% vs. 61.6%; Chi-square test, *χ*^2^ = 2.52; *df *= 1,* P* = 0.113), while the infestation intensity of *I. hexagonus*/*I. canisuga* for a subset of 209 co-infested hedgehogs was significantly higher than that of *I. ricinus* (mean of 24 vs. 11 ticks per co-infested animal; Wilcoxon signed rank test for paired data, *V* = 11663,* P* < 0.001). The highest recorded tick infestation intensity was 523 specimens (128 *I. ricinus*, 499 *I. hexagonus/I. canisuga*) in an adult female hedgehog, keeping in mind that occasionally not all ticks were collected.

**Table 2:** Prevalence and infestation intensities of ectoparasites in European hedgehogs (*n* = 498) in northern Germany

Prevalence and infestation intensities	Infested hedgehogs,* n*	Prevalence, %	Infestation intensity,* n*			
			Minimum	Maximum	Mean	Median
*Ticks*						
All tick species	431	86.5	1	523	27	9
*Ixodes ricinus* (total)	307	61.6	1	128	13	4
*Ixodes ricinus* (adults)	243	48.8	1	68	6	2
*Ixodes ricinus* (nymphs)	222	44.6	1	110	10	4
*Ixodes hexagonus/canisuga* (total)	332	66.7	1	499	23	6
*Ixodes hexagonus* (adults)	234	47.0	1	99	7	2.5
*Ixodes hexagonus/canisuga* (nymphs)	255	51.2	1	475	20	5
*Ixodes frontalis*	1	0.2	1	1	n.d	n.d
*Fleas*						
All flea species	455	91.4	1	1622	66	28
*Archaeopsylla erinacei*	455	91.4	1	1622	66	28
Ceratophyllidae	12	2.4	1	1	n.d	n.d
*Ceratophyllus sciurorum*	4	0.8	1	1	n.d	n.d
*Nosopsyllus fasciatus*	2	0.4	1	1	n.d	n.d
*Mites*						
*Caparinia tripilis*	88	17.7	n.d	n.d	n.d	n.d

*n.d.* Not determined

Regarding the monthly tick infestation rates, significant seasonal changes were observed only for *I. ricinus*, with high infestation rates from April to June (Table 3). In contrast, none of the considered variables had a significant effect on *I. hexagonus*/*I. canisuga* infestation probability, and the model did not explain the dataset any better than the respective null model.

**Table 3:** Results of the binomial generalised linear models testing the influence of sampling month, sampling year, sex, age, weight and health status on the infestation rates of European hedgehogs with *Ixodes ricinus* and *Ixodes hexagonus*/*I. canisuga*

Variables tested in GLM model	GLM Model A: *I. ricinus*				GLM Model B: *I. hexagonus*/*I. canisuga*			
	Estimate	SE	*Z*	*P*	Estimate	SE	*Z*	*P*
*Intercept*	− 0.25	0.96	− 0.26	0.797	0.62	0.96	0.65	0.517
*Sampling month (reference month: January)*								
February	0.40	0.76	0.53	0.598	0.14	0.77	0.19	0.851
March	0.73	0.74	0.99	0.320	0.17	0.74	0.23	0.818
April	2.46	0.72	3.43	0.001*	0.13	0.66	0.20	0.839
May	3.23	0.77	4.18	< 0.001*	0.14	0.65	0.22	0.829
June	2.09	0.75	2.78	0.005*	− 0.04	0.70	− 0.06	0.951
July	0.90	0.70	1.29	0.198	0.64	0.72	0.89	0.375
August	1.40	0.77	1.82	0.069	0.43	0.76	0.57	0.572
September	1.07	0.72	1.48	0.138	0.40	0.73	0.55	0.585
October	0.58	0.69	0.84	0.404	0.09	0.70	0.13	0.896
November	− 1.14	0.73	− 1.56	0.118	0.12	0.68	0.18	0.858
December	− 1.24	0.89	− 1.39	0.164	-0.65	0.77	− 0.85	0.397
*Sampling year (reference year: 2018)*								
2019	− 0.67	0.46	− 1.48	0.140	0.32	0.42	0.77	0.440
2020	− 0.74	0.46	− 1.60	0.110	0.06	0.43	0.15	0.882
2021	− 1.22	0.67	− 1.82	0.069	0.15	0.59	0.26	0.796
*Sex (reference: male)*	− 0.32	0.25	− 1.27	0.204	0.04	0.23	0.20	0.844
*Age (reference age: subadult)*	− 0.37	0.45	− 0.81	0.420	− 0.47	0.40	− 1.17	0.241
*Weight*	< 0.01	< 0.01	1.50	0.134	0.00	0.00	1.89	0.059
*Health Status (reference status: not impaired)*								
Moderately impaired	0.07	0.69	0.11	0.915	− 0.74	0.71	− 1.04	0.297
Severely impaired	0.01	0.70	0.01	0.989	− 0.70	0.72	− 0.97	0.333

*GLM *Generalised linear model, *SE* standard error

*Statistically significant at *P* ≤ 0.05

^a^Model A was significantly different from a null model: Chi-square = 107.23, *df* = 19, *P* < 0.001. Model B: Chi-square = 11.83, *df* = 19, *P* = 0.893

**Fig. 4:**
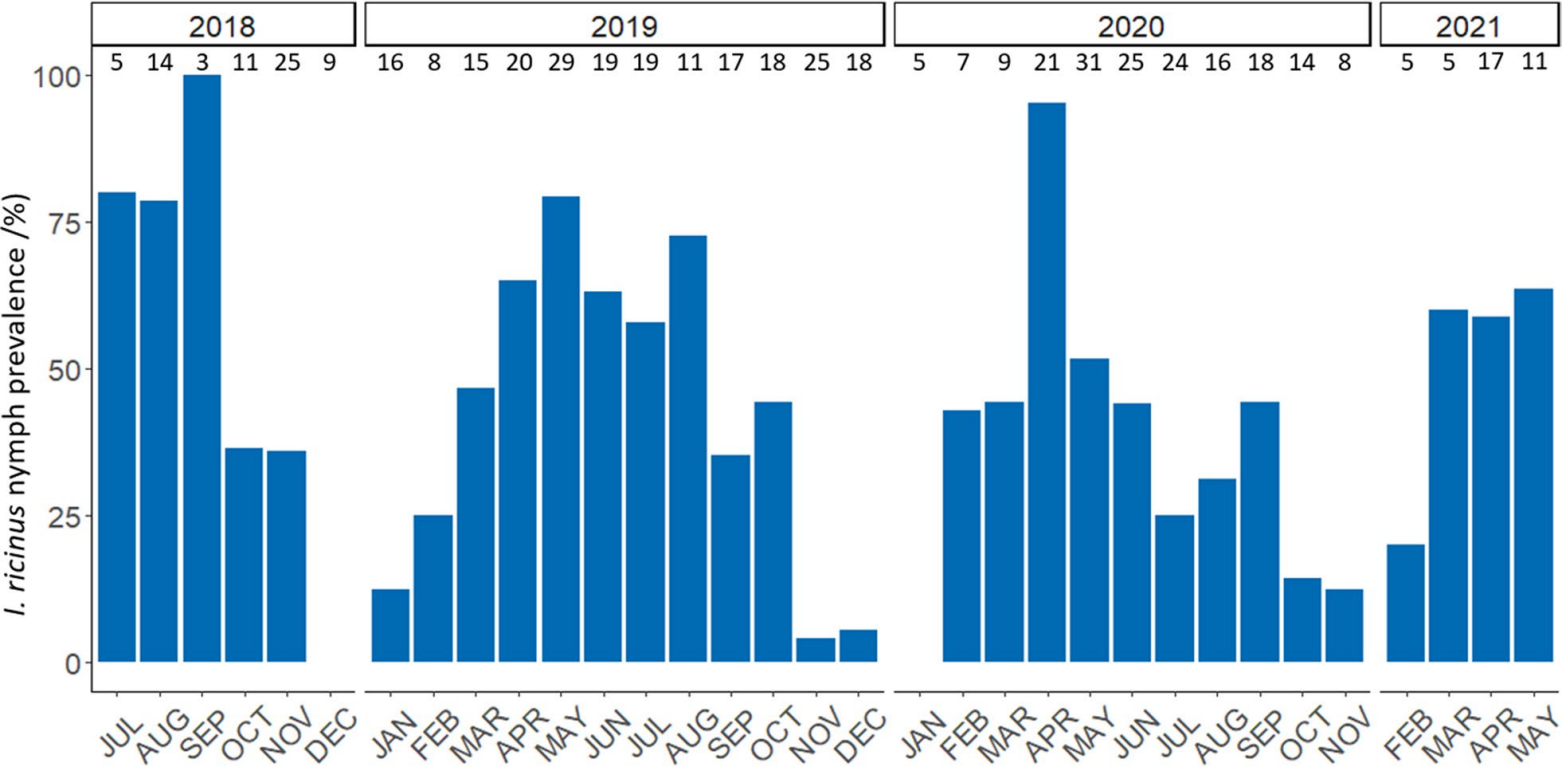
Prevalence of *Ixodes ricinus* nymphs on European hedgehogs in northern Germany during the study years 2018–2021. No animals were examined in the months December 2020 and January 2021

When adult and nymphal *I. ricinus* were considered separately, significantly higher infestation rates were evident for adult ticks from April to June, and for nymphal ticks from March to September, as compared to January (Table 4). Moreover, the prevalence of *I. ricinus* nymphs decreased significantly in study years 2020 and 2021 as compared to 2018 (Table 4; Fig. 4). While adult hedgehogs were significantly less often infested with adult *I. ricinus* than subadult hedgehogs, the prevalence increased with increasing body weight in both age categories. However, no significant relationship of age and body weight with the presence of *I. ricinus* nymphs was found (Table 4). In case of *I. hexagonus*/*I. canisuga*, the binomial GLMs for nymphs and adult ticks did not explain the dataset any better than the respective null model, indicating that none of the considered variables had a significant effect.

**Table 4:** Results of the binomial generalised linear models testing the influence of sampling month, sampling year, sex, age, weight and health status on the infestation rates of European hedgehogs with adult and nymphal *I. ricinus*

Variables tested in GLM model	GLM Model A: *I. ricinus* adults^a^				GLM Model B: *I. ricinus* nymphs^a^			
	Estimate	SE	*Z*	*P*	Estimate	SE	Z	*P*
*Intercept*	− 1.17	0.96	− 1.22	0.223	− 2.19	1.36	− 1.61	0.108
*Sampling month (reference month: January)*								
February	0.39	0.80	0.49	0.627	1.94	1.18	1.65	0.100
March	0.76	0.77	0.99	0.320	2.54	1.15	2.22	0.026*
April	1.99	0.70	2.83	0.005*	4.12	1.12	3.69	< 0.001*
May	2.50	0.71	3.50	< 0.001*	3.58	1.10	3.25	0.001*
June	2.03	0.75	2.71	0.007*	2.97	1.13	2.64	0.008*
July	0.92	0.72	1.27	0.205	2.29	1.12	2.04	0.041*
August	0.71	0.77	0.92	0.358	2.60	1.15	2.25	0.024*
September	0.47	0.75	0.63	0.531	2.49	1.13	2.20	0.028*
October	0.83	0.72	1.15	0.251	1.41	1.14	1.23	0.217
November	− 0.93	0.76	− 1.21	0.226	0.07	1.20	0.06	0.953
December	− 0.80	0.90	− 0.89	0.376	− 14.48	577.10	− 0.03	0.980
*Sampling year (reference year: 2018)*								
2019	− 0.43	0.42	− 1.02	0.310	− 0.92	0.48	− 1.92	0.055
2020	− 0.35	0.42	− 0.82	0.413	− 1.32	0.48	− 2.75	0.006*
2021	− 0.32	0.60	− 0.53	0.598	− 1.68	0.64	− 2.61	0.009*
*Sex (reference: male)*	− 0.20	0.23	− 0.86	0.392	− 0.33	0.24	− 1.38	0.168
*Age (reference age: subadult)*	− 0.89	0.42	− 2.13	0.033*	− 0.18	0.41	− 0.44	0.661
*Weight*	< 0.01	< 0.01	2.60	0.009	< 0.01	< 0.01	1.15	0.250
*Health status (reference status: health not impaired)*								
Moderately impaired	0.21	0.66	0.31	0.753	0.45	0.77	0.58	0.560
Severely impaired	< 0.01	0.67	< 0.01	0.999	0.49	0.77	0.64	0.525

*GLM *Generalised linear model, *SE* Standard Error

*Statistically significant at *P* ≤ 0.05

^a^Both models were significantly different from a null model. Model A: Chi-square = 79.25, *df* = 19, *P* < 0.001¸ Model B: Chi-square = 109.03, *df* = 19, *P* < 0.001

Regarding tick infestation intensity (tick species combined), no significant associations with the considered predictor variables were evident, except for lower infestation intensities in October and December (Table 5).

**Table 5:** Results of the generalised linear models testing the influence of sampling month, sampling year, sex, age, weight and health status on the number of ticks and number of fleas infesting European hedgehogs.

Variables tested in GLM model	GLM Model A: ticks^a^				GLM Model B: fleas^a^			
	Estimate	SE	*Z*	*P*	Estimate	SE	*Z*	*P*
*Intercept*	4.45	0.61	7.25	< 0.001*	5.37	0.56	9.60	< 0.001*
*Sampling month (reference month: January)*								
February	0.11	0.52	0.21	0.834	-0.37	0.48	-0.77	0.440
March	− 0.56	0.51	− 1.11	0.268	0.22	0.46	0.48	0.635
April	− 0.20	0.45	− 0.45	0.653	− 0.99	0.41	− 2.39	0.017*
May	0.02	0.44	0.05	0.962	− 1.23	0.41	− 3.03	0.002*
June	− 0.52	0.48	− 1.08	0.282	− 0.91	0.44	− 2.08	0.038*
July	− 0.21	0.47	− 0.44	0.662	− 1.05	0.43	− 2.42	0.016*
August	− 0.39	0.50	− 0.77	0.441	− 1.13	0.46	− 2.46	0.014*
September	− 0.62	0.49	− 1.27	0.203	− 1.19	0.45	− 2.65	0.008*
October	− 1.46	0.48	− 3.06	0.002*	− 0.01	0.43	− 0.03	0.975
November	− 0.56	0.46	− 1.21	0.228	− 0.09	0.42	− 0.22	0.828
December	− 1.79	0.54	− 3.29	0.001*	0.07	0.49	0.13	0.894
*Sampling year (reference year: 2018)*								
2019	− 0.32	0.28	− 1.15	0.251	− 0.26	0.25	− 1.05	0.295
2020	− 0.52	0.28	− 1.85	0.065	− 0.39	0.26	− 1.51	0.130
2021	− 0.70	0.39	− 1.78	0.074	− 0.54	0.36	− 1.49	0.137
*Sex (reference: male)*	− 0.08	0.15	− 0.54	0.591	− 0.25	0.14	− 1.78	0.075
*Age (reference: subadult)*	0.47	0.27	1.75	0.081	1.03	0.24	4.22	< 0.001*
*Weight*	0.00	0.00	− 1.12	0.262	0.00	< 0.01	− 1.97	0.049*
*Health status (reference status: not impaired)*								
Moderately impaired	− 0.55	0.42	− 1.31	0.191	− 0.71	0.39	− 1.83	0.067
Severely impaired	− 0.43	0.43	− 0.99	0.324	− 0.47	0.39	− 1.18	0.237

*GLM *Generalised linear model, *SE* standard Error

*Statistically significant at *P* ≤ 0.05

^a^Both models were significantly different from a null model. Model A: Chi-square = 38.0, *df* = 19, *P* = 0.006. Model B: Chi-square = 68.6, *df* = 19, *P* < 0.001

### Fleas

In all cases of flea infestation, *A. erinacei* was identified. Additionally, 12 hedgehogs each carried one flea of the family Ceratophyllidae. Sequencing of the partial COX-2 gene identified *Ceratophyllus sciurorum* (reference sequence: GenBank accession no. MG637398; identity: 100%, query cover: 98%) in four hedgehogs, while two hedgehogs carried *Nosopsyllus fasciatus* (reference sequences: acc. nos. LR991721, MG637390, HQ881597; identity: 97.9–99.2%, query cover 98–100%). Generated sequences were deposited at GenBank under accession numbers OR666981-OR666982 (*N. fasciatus*) and OR666983-OR666986 (*C. sciurorum*). In the remaining six cases, the fleas could not be differentiated due to non-amplification (*n* = 4) or poor sequences (*n* = 2). Moreover, one specimen of *Ctenocephalides felis* was found on one hedgehog.

**Fig. 5:**
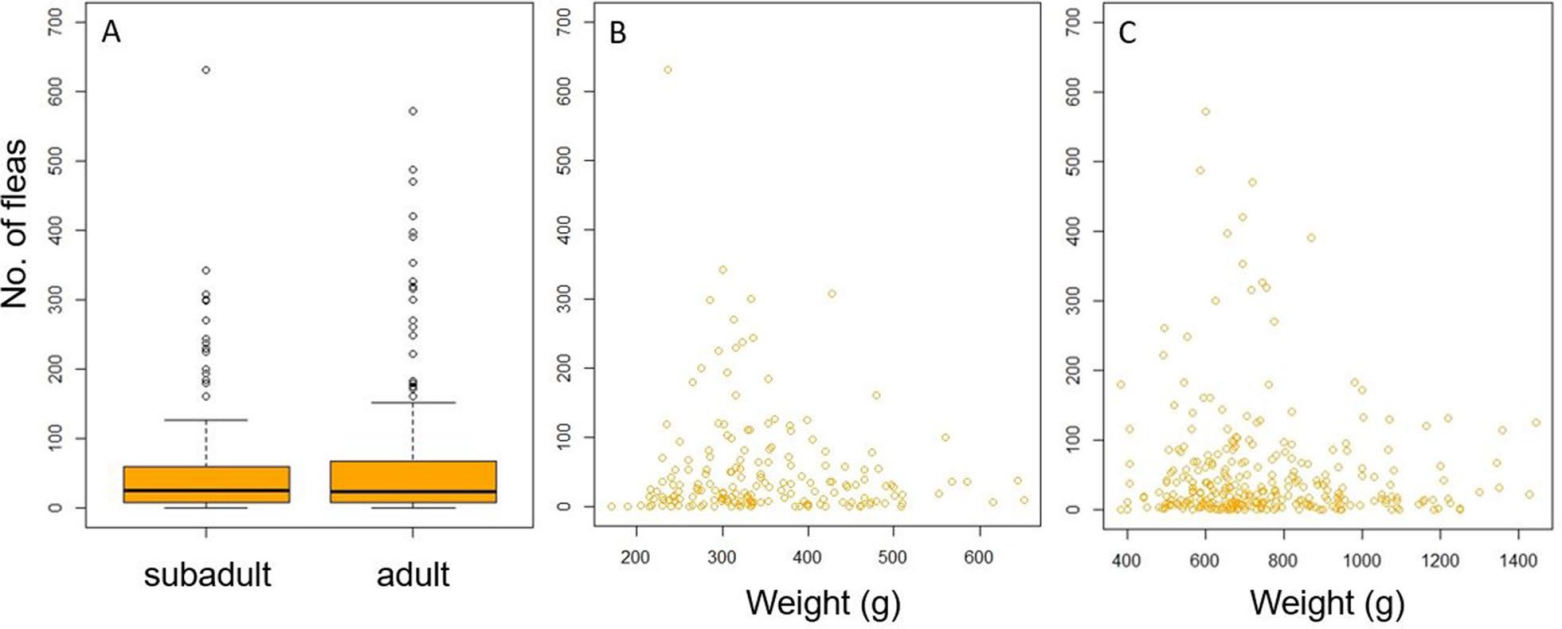
Boxplot of flea infestation intensity according to the age of the hedgehogs (**a**), weight of subadult hedgehogs (**b**) and weight of adult hedgehogs (**c**)

Due to the high flea prevalence (91.4%), no binomial GLMs for flea presence/absence were calculated. The maximal infestation intensity with *A. erinacei* amounted to 1622 fleas on one adult male hedgehog as estimated from the weight of the collected fleas, with a mean infestation intensity of 66 fleas per animal. Significantly lower infestation intensities were found during the months April to September. Furthermore, the flea burden was significantly higher on adult than subadult hedgehogs, although the number of fleas decreased with an increasing body weight in both age categories (Fig. 5; Table 5). No significant correlation of flea burden with the health status of the animals was detected (Table 5).

### Mites

**Fig. 6:**
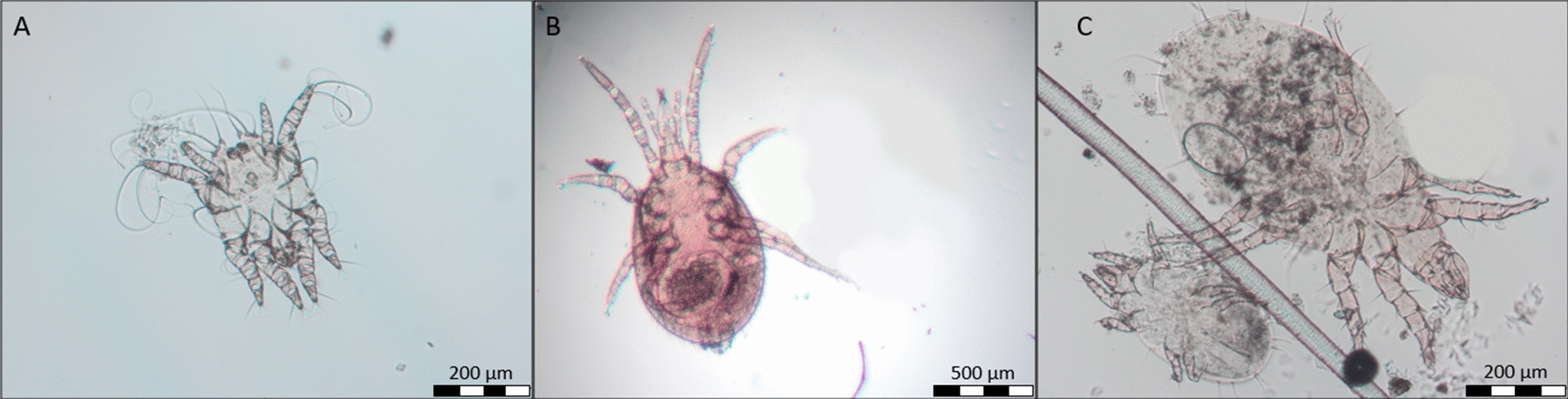
Male specimen of *Caparinia tripilis* (**a**), mite of the family Macronyssidae (**b**) and free-living mite of the family Acaridae (**c**) recovered from European hedgehogs in northern Germany

In all mite-infested hedgehogs, *C. tripilis* was detected (Fig. 6). Additionally, one sample contained free-living mites, most probably members of the family Acaridae (Fig. 6).

The prevalence of mite infestation was significantly lower during the month of February as well as from May to October as compared to January. Moreover, the GLM showed that adult hedgehogs were significantly more often infested by mites than subadult animals (Table 6). No correlation was evident regarding the health status of the animals.

**Table 6:** Results of the binomial GLM testing the influence of sampling month, sampling year, sex, age, weight and health status on the infestation rate of European hedgehogs with the mite *Caparinia tripilis*

Variables tested in GLM model	GLM model^a^			
	Estimate	SE	*Z*	*P*
*Intercept*	− 17.01	968.50	− 0.02	0.986
*Sampling month (reference month: January)*				
February	− 2.34	1.17	− 2.00	0.045*
March	0.05	0.94	0.06	0.954
April	− 0.76	0.89	− 0.85	0.393
May	− 1.92	0.90	− 2.14	0.032*
June	− 2.42	0.95	− 2.53	0.011*
July	− 2.44	0.96	− 2.55	0.011*
August	− 2.09	0.96	− 2.17	0.030*
September	− 2.86	1.04	− 2.76	0.006*
October	− 2.86	1.12	− 2.55	0.011*
November	− 1.40	1.05	− 1.34	0.182
December	0.71	1.08	0.66	0.510
*Sampling year (reference year: 2018)*				
2019	0.51	0.74	0.68	0.494
2020	1.36	0.73	1.85	0.065
2021	1.41	0.88	1.62	0.106
*Sex (reference: male)*	− 0.47	0.30	− 1.56	0.120
*Age (reference age: subadult)*	2.16	0.62	3.50	< 0.001*
*Weight*	0.00	< 0.01	− 0.12	0.902
*Health status (reference status: not impaired)*				
Moderately impaired	14.48	968.50	0.02	0.988
Severely impaired	15.14	968.50	0.02	0.988

*GLM* Generalised linear model, *SE* Standard Error

*Statistically significant at *P* ≤ 0.05

^a^The GLM model was significantly different from a null model (Chi-square = 80.7, *df* = 19, *P* < 0.001)

One adult female hedgehog, which was possibly pre-treated against ectoparasites and therefore not included in the data set of 498 hedgehogs described in this paper, should be mentioned here also, as it was additionally infested by three specimens of the family Macronyssidae, possibly *Ornithonyssus bacoti* (Fig. 6), in addition to fleas. However, these mites of the family Macronyssidae could not be unambiguously identified as the anal plate was not well visible, and molecular analysis was not possible as all specimens were slide-mounted.

## Discussion

With an infestion rate of 98.6%, almost all of the European hedgehogs examined in this study were infested with at least one ectoparasite species. This finding confirms the common knowledge that hedgehogs are highly susceptible to ectoparasite infestation and thus may pose a threat to humans in terms of zoonotic disease transmission. The predominant ectoparasites identified were hedgehog-specific species such as *I. hexagonus*, *A. erinacei* and *C. tripilis*, as well as the generalist tick species *I. ricinus*.

### Tick infestations

In previous studies on European hedgehogs, the most common tick species was *I. hexagonus* [11, 19, 41, 42]. In the UK, Gaglio et al. [1] detected only *I. hexagonus* when examining dead hedgehogs. In the present study, however, the generalist tick species *I. ricinus* showed nearly the same prevalence as *I. hexagonus*, and Földvári et al. [2] even found *I. ricinus* more often than *I. hexagonus* on Northern white-breasted hedgehogs in Budapest, Hungary. Moreover, co-infection with both *Ixodes* species was common in the hedgehogs examined in the present study.

Unfortunately, it was not possible to differentiate the nymphs and larvae of *I. hexagonus* and *I. canisuga* due to their high degree of morphological similarity. But as no adults of *I. canisuga* were found on the hedgehogs, it is likely that the nymphs and larvae, which could not be morphologically determined as *I. canisuga* or *I. hexagonus,* belonged to the latter species. To the authors’ knowledge, so far *I. canisuga* has not been identified on European hedgehogs in Germany, although a report exists from Ireland [43]; thus, this hedgehog species can be infested by *I. canisuga*.

To our knowledge, the present study reports the first detection of *I. frontalis* on a European hedgehog. This ornithophilic tick species is found both on birds and in their nests, but is sometimes also detected by the flagging method [44]. As only one hedgehog was infested by a single *I. frontalis,* this was probably an accidental infestation. Possible sources of infestation can be contact with questing specimens (e.g. fallen off a bird nest in trees), bird nests on the ground or dead birds. As hedgehogs are known to feed on eggs or carrion when food availability is low, this could explain the infestation with *I. frontalis*.

Regarding seasonal changes in tick infestation, we noted different infestation patterns for *I. hexagonus* and *I. ricinus* ticks. For *I. hexagonus,* no significant seasonal changes in infestation rates were evident, although a spring and autumn peak in infestations has been described by Arthur [45]. This described spring activity peak of *I. ricinus* [46] was reflected in the present study, but no distinct autumn peak was observed regarding the infestations of hedgehogs. Infestation probability with adult *I. ricinus* was highest in April, May and June, while nymphs of *I. ricinus* showed higher infestation rates throughout the warmer period from March until September, particularly in April and May. Furthermore, both tick species were found on hedgehogs during the winter months. For *I. hexagonus*, winter activity was reported in earlier studies by Liebisch et al. [41] and Arthur [45]. As an endophilic tick species, *I. hexagonus* is less affected by temperature changes than the exophilic species *I. ricinus*, thereby facilitating a year-round activity and constant infestation rates throughout the year, as also described by Probst et al. [47] for dogs and cats in Germany. These authors also showed winter activity of *I. ricinus* in Germany [47], which was confirmed in the present study by the occurrence of *I. ricinus* on hedgehogs throughout the year. Taking into account that hedgehogs hibernate during the cold winter months, this finding is even more interesting because they should have no contact to *I. ricinus* during hibernation. Nevertheless, as the study animals were found due to a health problem, they were active despite the hibernation period.

Regarding tick infestation rates over the study years, we noted a significant decline in *I. ricinus* nymph prevalence on hedgehogs in 2020 and 2021. Similarly, a continuous decline of questing *I. ricinus* density was observed in the urban area of Hanover from 2018 to 2022 via the flagging method [48]. An explanation for this decline may be climatic changes. The period of 2018–2020 covered the warmest years since the start of systematic weather data collection in Germany in 1881 and these years were additionally characterised by less than average rainfall [49]. As ticks are sensitive to desiccation, this drought during these years may have negatively affected their population density. The fact that this decline was only seen in nymphs and not in adult ticks may be related to the fact that *I. ricinus* larvae are more sensitive to desiccation than the nymphal and adult stages, as already shown for the northern American blacklegged tick *Ixodes scapularis* [50, 51]. Since moulting and the beginning of host-seeking activity of the nymph emerging from the larva require a considerable period of time, the decline caused by the exceptional drought in 2018 and the years thereafter becomes apparent with a time delay, resulting in statistically significant reductions in nymph prevalence as of 2020.

Hedgehog body weight showed a significant positive correlation with the infestation probability with adult *I. ricinus*. This may be due to a higher level of activity, as presumably animals with a higher body weight are healthier and thus more active. As *I. ricinus* is an exophilic tick, animals must leave their nest to become infested. Interestingly, the statistical model showed that adult hedgehogs were significantly less often infested by adult *I. ricinus* than subadult hedgehogs. Adult hedgehogs brought to rescue centres are often sick and may have an even lower body weight than subadult animals, thus are less active and at a reduced risk of acquiring *I. ricinus* ticks. Dudek et al. [52] noted that larger hedgehogs carried more ectoparasites (ticks and fleas) than smaller individuals, without taking into consideration the age of the animals. Notably, the mentioned study took the body length and not the body weight into account.

### Flea infestations

As reported in numerous previous studies [1, 11, 13, 20], the majority of fleas collected from hedgehogs in the present study were identified as *A. erinacei,* confirming this as the most important flea species infesting hedgehogs. Only three other flea species were detected. The prevalence of Ceratophyllidae was very low, with only 12 infested hedgehogs, each of which carried only one specimen. *Ceratopsyllus sciurorum* mainly infests squirrels, but it has also been found on different mammals, even posing a problem in mink farms [53]. This flea species is of concern as a vector of canine distemper virus [54], which is a dangerous pathogen for different canids and mustelids and thus also poses a high risk for pet animals. Furthermore, the rat flea *N. fasciatus* usually infests rodents, although it can spread to other mammalian species, for example through predation, and it is also known to infest humans [34]. Both squirrels and rats are common inhabitants of urban gardens and parks. Rats in particular, which are often found at hedgehog feeding stations, might come into close contact with the study species, resulting in ectoparasite transmission. In addition to Ceratophyllidae, only one specimen of *C. felis* was found on one hedgehog. This was surprising, as the cat flea is a very common ectoparasite in different mammals [55, 56]. Moreover, hedgehogs share the same habitat with cats in urban areas, living predominantly in gardens. Therefore, a higher infestation rate of hedgehogs with *C. felis* was expected.

Seasonal examination showed significantly lower *A. erinacei* burdens in summer. The mating season of European hedgehogs starts in May and results in a high activity, especially of male hedgehogs searching for a mate. Additionally, hedgehogs use many different daytime nests in summer. They might even rest in a place that has not been used before and is not as enclosed as a hibernating or breeding nest. As *A. erinacei* is a nest-adapted flea species, it may be more difficult for this flea species to find a host during the summer months. Higher flea infestation intensities from October going forward might also be explained by hedgehogs spending more time resting in their nests and thus having more contact with *A. erinacei*. Moreover, Brinck et al. [57] assumed that the hedgehog flea primarily reproduces in nests of female hedgehogs rearing their young, which may result in high levels of flea infestation in hoglets, which are leaving the nests in September and October, as well as in female hedgehogs. Thus, the infestation level with fleas increases from October on. However, in the present study infestation intensity with fleas was higher in adult than in subadult hedgehogs and no sex difference was observed. This age pattern is similar to the findings of Dudek et al. [52], who noted higher tick and flea burdens on larger hedgehogs in terms of body length. In addition, in the present study, the flea number was negatively correlated with the body weight of the hedgehogs in both age categories. There might be a diluting effect, as the flea burden of one breeding nest is distributed on several animals, resulting in hoglets not being more infested than adult animals. As young hedgehogs need more time to increase their weight prior to hibernation than adult animals, they are active later in the year. Those subadult hedgehogs having weight or health problems might even still be active in December and January and are then brought into rehabilitation centres for medical care. This might explain that the highest flea burdens were detected in January.

### Mite infestations

*Caparinia tripilis* was the major mite species detected, as in previous studies [13, 26, 27, 37]. However, it should be kept in mind that only mites dwelling on the skin surface were examined in the present study, as skin scrapings were not taken due to ethical reasons. Thus, mite species such as *Sarcoptes scabiei*, *Notoedres cati* and *Demodex erinacei* were probably missed. The detected Macronyssidae mites may have been specimens of the tropical rat mite *O. bacoti,* which is a blood-feeding mite of zoonotic importance. This species has been previously detected in European hedgehogs [13] as well as in African pygmy hedgehogs (*Atelerix albiventris*) [58]. It is not host specific, thus, it can infest multiple hosts, including humans [59, 60]. Mites of the family Acaridae were also detected; these mites are considered to be free-living mite species without medical importance. In the present study, only single specimens were found, but in cases of high infestation they can lead to dermatitis or contact allergy [37].

Mite infestation rates varied seasonally, in part driven by the different ratio of adult to subadult hedgehogs examined. Adult animals were significantly more often infested by mites, possibly due to adult hedgehogs being often in a bad health status and thus being predisposed to mite infestation. In addition, adult animals have probably been infested for a longer period than subadult hedgehogs, which is presumably associated with a higher infestation intensity and can therefore be detected more easily. In both age classes, significantly lower mite infestation rates were observed in February and during the summer months as compared to January. February is one of the coldest months of the year in Germany, with a mean air temperature of - 1 °C to 6 °C in February of 2018–2021 [61]. Thus, the cold temperature might be an explanation for the low infestation rates, not only because hedgehogs are less active in the cold, but also due to a slower development of mites during colder climatic conditions which is known for several mite species [62–64]. The infestation rate increased in March, when hedgehogs start to awake from hibernation and temperatures rise, so that mite infestation can grow rapidly. Moreover, more adult hedgehogs were examined in March as compared to February, which had a significantly higher probability of mite infestation, contributing to the sharp prevalence increase. In summer, infestation rates decreased again. Higher infestation rates in winter than in summer were also described by Brockie [65], who explained this finding by the bad health status of hedgehogs with mite infestation, preventing them from going into or rousing them from hibernation. The same was probably the case in the present study, i.e. healthy hedgehogs were hibernating and thus not examined, resulting in seasonal overestimation of the mite infestation rate.

### Health impairment

Previous studies showed a correlation between the health status of hedgehogs and their ectoparasites, such as, for example, a regenerative anemia due to tick infestation [66]. Furthermore, *I. hexagonus* is attracted by the faecal odour of sick hedgehogs, potentially leading to a higher tick infestation of sick animals compared to healthy ones [67]. A correlation between mite infestation and weakness as well as abnormal daytime activity of hedgehogs has also been suggested [65]. However, no significant correlation between the health status category and ectoparasite presence or ectoparasite intensity was detected in the present study. As a caveat, no differentiation between chronic and acute disease, such as injury, was made in the present study, possibly explaining this result. Furthermore, additional factors may influence the ectoparasite load of animals, such as whether the particular hedgehog’s territory sustains large tick populations [11]. Despite the absence of a correlation with the health status category, hedgehogs with a lower body weight carried more fleas, and adult hedgehogs had higher flea burdens and a higher mite infestation probability than subadults. Adult hedgehogs also showed a severely impaired health status more often than subadult hedgehogs (68.0% vs. 29.6%). In particular, adult hedgehogs with a lower weight usually have an underlying health problem, but also subadult hedgehogs, which did not develop normally and are thus often smaller, might have an impaired immune system. In contrast, tick burden did not show a significant relationship with age or body weight, although the mean tick count was highest in severely impaired animals. A significant relationship was only evident regarding the probability of infestation with adult *I. ricinus*, which was lower in adult animals as well as in animals with a lower body weight. Therefore, there is more likely to be a correlation between an impaired health status and a flea infestation than a tick infestation.

In the light of the present study, ectoparasite treatment is of major importance when taking care of hedgehogs, not only to minimise negative effects on the host but also to decrease the risk of pathogen transmission to other animals and humans. In the case of tick infestation, it is best to remove the ticks manually using a forceps or tick remover. Further, ectoparasitic sprays containing, for example, propoxur can be used in case of flea infestation and injection or topical treatment with, for example, ivermectin, doramectin, fluralaner or phoxim in case of mite infestation [68–70]. Additional treatment to support the skin, fur and spine regeneration during a mite treatment (e.g. vitamin, zinc and essential fatty acids supplementation) is recommended.

## Conclusions

High ectoparasite prevalences and infestation intensities were recorded in European hedgehogs presented at rehabilitation centres in the present study, with some seasonal variation throughout the year. In addition, a most likely climate change-driven decline in *I. ricinus* nymph prevalence due to drought years was observed as of 2020. Although no statistically significant association of ectoparasite infestation with the animals’ health status was apparent, infestation intensity of fleas and infestation rates of mites were significantly higher in adult hedgehogs, which were more often in a severely impaired health status. Moreover, animals with a lower body weight, which can be indicative of a suboptimal condition, had a higher flea infestation intensity. In conclusion, hedgehogs taken into human care should be immediately treated against ectoparasites, also in light of the vector function of the encountered ectoparasite species. Their high ectoparasite infestation rate may predispose hedgehogs to serve as reservoirs of zoonotic vector-borne diseases. In the frame of the One Health concept, the epidemiological relevance of this synanthropic species for the circulation of vector-borne pathogens should thus be further investigated.

## Data Availability

Data supporting reported results is contained within the article. Generated sequences were deposited at GenBank under accession nos. OR666981-OR666982 (*N. fasciatus*) and OR666983-OR666986 (*C. sciurorum*).

## Abbreviations

BLAST: Basic local alignment search tool
COX-2: Cytochrome c oxidase subunit 2
GLM: Generalised linear model
NCBI: National Centre for Biotechnology Information
